# Hospital Clerks: Their Duties and Responsibilities.—II

**Published:** 1912-01-20

**Authors:** Herbert J. Dafforne

**Affiliations:** Chief Clerk Leicester Infirmary.


### HOSPITAL OFFICERS: THEIR TRAINING AND WORK.

### Hospital Clerks: Their Duties and Responsibilities.—II.*

By HERBERT J. DAFFORNE, Chief Clerk Leicester Infirmary.

The Accountant Clerk.

As an accounts clerk his duties are to keep all the accounts, for which purpose he must have a thorough knowledge of book-keeping and have the uniform system of hospital accounts at his finger ends. He must see that all moneys received are paid into the Bank, that receipts are properly made out and forwarded, and that his cash-analysis book is properly entered up and agrees with the bankers' pass-book. He must analyse all invoices, making sure the goods charged for have been received, that the price charged is correct, and that the extensions have been properly made and added up. When this has been done he prepares them for the committee for payment, entering them through his various books first. He must have his books ready for audit at any time, and see that all vouchers are present. At the close of the year it falls to his duty to obtain the figures of income and expenditure for the whole year, and to present them in the Uniform System of Hospital Accounts to the secretary for the board.

The Senior Clerk.

The chief or senior clerk should have been through each of the previous appointments, so that he may have a thorough knowledge of every detail of his office. His duties are generally to relieve the secretary of as much clerical and routine work as possible, so as to enable him to devote his whole time and energy to the general management of the institution, and to the building up of its income on a sound financial basis. He must compile the annual report—no light task—and keep an accurate list of all subscriptions paid or unpaid. To do this effectively the card system of filing is as good as any, for in this each individual, firm, or body of people subscribing has a separate card, on which any remarks or notes peculiar to them can be made. The senior clerk must prepare all necessary statistics, conduct parties round the institution and interest them in the work, take his chief's place when absent, answer correspondence, take board and committee meetings, and, if necessary, obey the call of propaganda, and go out occasionally to describe the work of the institution to Friendly Society demonstrations, adult schools, and such-like gatherings. Then it may be his duty to discharge in-patients, after ascertaining that they are quite satisfied with the treatment they have received, and he also may have to keep these patients' records systematically for future reference. He must order all printing and stationery required, and also any other goods which come under the jurisdiction of the secretary's department.

A Final Hint.

The foregoing is a rough outline of a progressive hospital clerk's multifarious duties, but it largely depends on the size of the institution whether these appointments are divided amongst two, three, or perhaps four clerks, for in very large hospitals it may be necessary for two or three clerks to do the work connected with one department. Where possible, it is a good thing for a hospital clerk to act as steward's clerk for a short period, as by this means he obtains a very useful and sound knowledge of the catering and maintenance necessary to a large institution.

The small paragraph which appeared in The Hospital of November 25, 1911, should be well remembered by each clerk engaged in hospital work, namely, "In the voluntary hospital the claims of the patient, his needs, and adequate protection are the first considerations with all who are responsible for its administration." By always bearing this in mind the clerk in his limited sphere helps to create public confidence in his institution, which goes a long way to encourage its supporters to continue and increase their help.

(*Continued from our issue of January* 6, *p.* 369.)

